# Rapid, multiplex detection of SARS-CoV-2 using isothermal amplification coupled with CRISPR-Cas12a

**DOI:** 10.1038/s41598-022-27133-7

**Published:** 2023-01-16

**Authors:** Diogo Figueiredo, António Cascalheira, Joao Goncalves

**Affiliations:** 1 Carbus, Bioengineering and Nanosystems (IST), Lisboa, Portugal; 2 Carbus, Lisboa, Portugal; 3 iMed- Research Institute for Medicines, Faculdade Farmácia da Universidade Lisboa, Lisboa, Portugal

**Keywords:** Biochemistry, Biological techniques, Biotechnology, Microbiology

## Abstract

In December 2019 an outbreak erupted due to the beta coronavirus Severe Acute Respiratory Syndrome Coronavirus 2 in Wuhan, China. The disease caused by this virus (COVID-19) rapidly spread to all parts of the globe leading to a global pandemic. Efforts to combat the pandemic rely on RT-qPCR diagnostic tests that have high turnaround times (~ 24 h), are easily contaminated, need specialized equipment, facilities, and personnel that end up increasing the overall costs of this method. Loop-mediated isothermal amplification (LAMP) coupled with a reverse transcription step (RT-LAMP) is an alternative diagnostic method that can easily overcome these obstacles, when coupled with CRISPR/Cas it can eliminate false positives. Here we report a fast (~ 40 min), highly sensitive, point-of-care multiplex RT-LAMP and CRISPR/Cas12a assay to detect SARS-CoV-2. This fluorescence-based test achieved 100% specificity and 93% sensitivity using 25 positives and 50 negative patient samples for Ct < 35. Our reported LoD of 3 copies/µL will enable the robust, fast detection of the virus in a dedicated equipment which is a major step towards population-wide accessible testing.

## Introduction

The 2019 outbreak of Severe Acute Respiratory Syndrome Coronavirus 2 (SARS-CoV-2) that began in Wuhan China and led to a global pandemic caused a massive overload of health systems. The disease called COVID-19 rapidly spread to the entire globe, being transmitted by droplets that contain high viral loads capable of infecting an individual. As of 21 of September 2021, there are a total of 228 million infections worldwide with a death toll of 4.7 million people (WHO).

For the diagnostic of viral agents, nucleic acid amplification tests (NAAT) are the most used diagnostic tool and have been extensively reported during past outbreaks of different coronavirus such as Middle East Respiratory Syndrome (MERS) and Severe Acute Respiratory Syndrome Coronavirus (SARS-CoV). The current “gold standard” to detect active SARS-CoV-2 infections is Reverse-Transcription Quantitative Polymerase Chain Reaction (RT-qPCR). This technique has a high efficiency while generating a large number of amplicons (~ 10^9^)^1,2^. In a low-resource environment these types of tests are inaccessible, there is thus a need to develop affordable and point-of-care diagnostic tools to provide low- and mid- income countries with the necessary tools to accurately track infections. To remove the need of using a thermocycler, isothermal nucleic acid amplification methods were the first choice, the constant temperature of the reaction and short amplification times allow for the miniaturization of the equipment and a more point-of-care approach^3,4^

Loop-mediated isothermal amplification (LAMP) attracted more attention. This technique can be coupled to a reverse transcription step with colorimetric, turbidity and/or fluorescence-based detection of amplicons^5–7^. It is conducted between 60 and 65 °C and uses 4 to 6 primers (PCR only uses 2) complementary to different regions within the analysed DNA, in combination with different DNA polymerases that have strand displacement activity. Multiple tests have been developed using this method, to detect infectious agents such as Zika, influenza, tuberculosis, and malaria^7–9^. A number of different tests using this method have also been developed for the detection of SARS-CoV-2^10–16^. These usually detect 1 or 2 genes simultaneously and thus, much like RT-qPCR, these might miss positive results that did not have amplification in one of these genes (or both). The optimal detection system would need to detect at least 3 different targets at the same time to erase all doubt regarding the final result. Despite being an extremely fast amplification method (around 30 min), or even due to this, this method is associated with a high propensity to generate false positive results. To tackle this problem, new diagnostic technologies combining this method with CRISPR/Cas are being developed^17–20^.

To tackle the false positive problem several innovative solutions have been developed mainly using RNA-guided CRISPR/Cas detection of nucleic acids since it allows for high sensitivity, specificity, and reliability. The basis of this technology is using a Cas nuclease associated with a specific guide RNA (gRNA) that will determine the target sequence of the effector protein. Once this protein is activated (target sequence is present) it will cleave surrounding single-strand nucleic acids indiscriminately (this activity is called collateral cleavage) due to its intrinsic trans nuclease activity. This activity can enable nucleic acid detection by using guide sequences specific for the product of the isothermal amplification, leading to the cleavage of an F–Q reporter enabling fluorescence-based detection of the viral genetic material^21–23^.A number of different CRISPR/Cas diagnostic tests have been reported, Table 1 shows a summary of some of the most relevant ones. Some of these diagnostic methods do not use an internal control to verify the integrity of the sample, this is a crucial point as there might still be remnant virus RNA in cells after an infection but, the individual does not pose a health hazard to others. This might lead to incorrect assessment regarding the infectivity of the person (which also happens with RT-PCR due to its very high sensitivity) which can cause congestion of diagnostic centres and/or hospitals.

**Table 1:** Comparison between different CRISPR/Cas based diagnostic methods. COVsense (this study); iSCAN^15^; opvCRISPR^16^; SHERLOCK^26^; DETECTR^17^; CDC Assay^27^.

	COVsense	iSCAN	opvCRISPR	SHERLOCK	DETECTR	CDC assay
Target gene	E/N/ORF1a	E/N	S	S/ORF1ab	N/E	N
Control gene	Rnase P	Rnase P	–	Rnase P	Rnase P	Rnase P
Total assay time	40 min	1 h	45 min	1 h	1 h	1.5 h
Nucleic acid extraction	No need	Yes	Yes	No need	Yes	Yes
Amplification type	RT-LAMP	RT-LAMP	RT-LAMP	RT-RPA	RT-RPA	RT-PCR
Enzyme type	LbCas12a	LbCas12a	LbCas12a	LwCas13a	LbCas12a	–
Quantitative	No	No	No	No	No	Yes
LOD (copies per reaction)	3	10	5	10	10	1
Readout type	FL	FL/LF	FL	LF	FL	FL
Specificity	100%	100%	100%	98.5%	100%	100%
Sensitivity	93%	94%	100%	93%	100%	100%
Sample size	75 (50)	48 (12)	50 (24)	402 (200)	28 (20)	117(104)

() indicate negative samples*.*

*E* envelope protein gene, *N* nucleocapsid protein gene, *ORF1a* open reading frame 1a gene, *S* spike protein gene. *RT* reverse transcription, *LAMP* loop mediated isothermal amplification, *RPA* recombinase polymerase amplification, *PCR* polymerase chain reaction. *FL* fluorescence, *LF* lateral flow.

Thus, we believe that by using an internal control to provide information regarding the sample integrity (Rnase P) and the simultaneous detection of 3 different target genes in the viral genome it is possible to avoid false positives, correctly assess the infectivity of the individual while still retaining a fast sample to result time. This system was then integrated onto a dedicated proof of concept device that can be easily transported and thus suitable for point of care testing.

## Results and discussion

### Detection of SARS-CoV-2 by RT-LAMP

#### Commercially available open-source kits.

**Figure 1:**
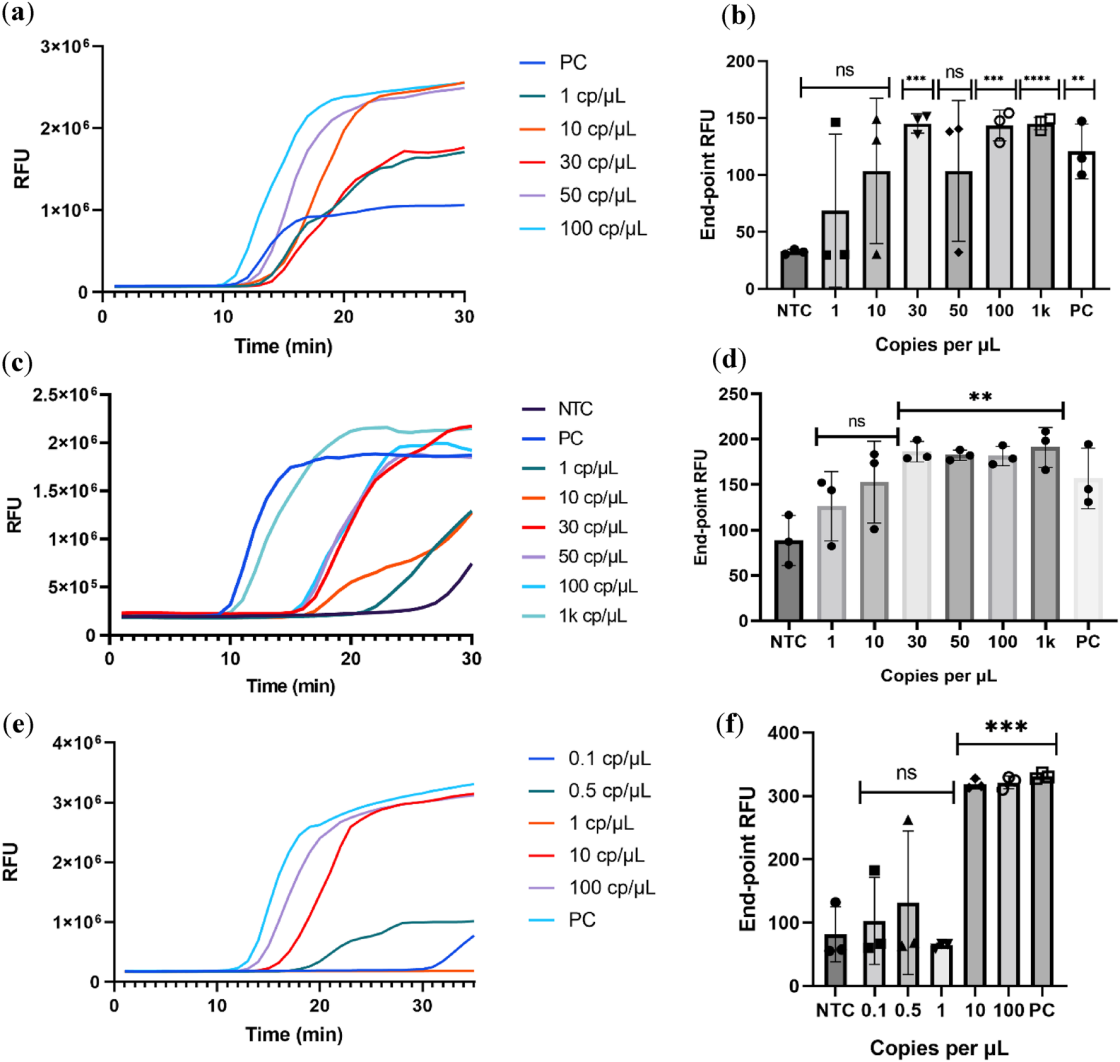
Real-time fluorescence for the NEB RT-LAMP reactions using the N/E primer set (**a**), the As1 primer set (**c**) and the N/E/As1 combined primer set (**e**). End point fluorescence measurements for the respective primer sets (**b**), (**d**) and (**f**). Statistical significance was determined by unpaired two-tailed t-test and all data were shown as mean ± S.D of 3 replicates. Asterisks indicate **P < 0.01; ***P < 0.001; ****P < 0.0001 and “ns” is non-significant.

**Figure 2:**
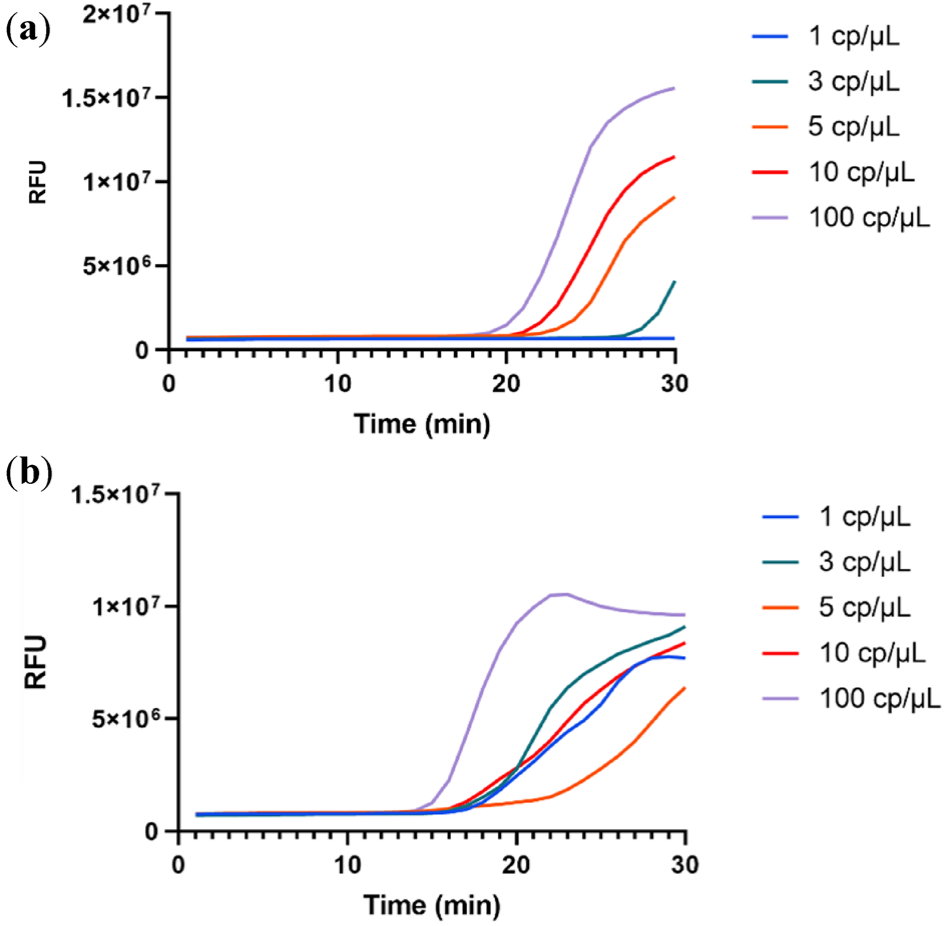
(**a**) Real-time fluorescence signal for the amplification of synthetic RNA controls using a 3-primer set with 40 mM of Guanidine HCL in the RT-LAMP reaction. (**b**) Amplification of synthetic RNA controls using a 3-primer set with 40 mM of Guanidine HCL and extra Bst 2.0 polymerase.

It is possible to achieve very high sensitivity by combining isothermal amplification methods (RT-LAMP) with the simultaneous detection of different target sites on the SARS-CoV-2 genome by the CRISPR/Cas12 system. When detecting SARS-CoV-2 via RT-LAMP, we found that commercially available kits could only achieve a significant response to 30 copies/µL, high propensity to produce false positive results and recurrent on/off behaviour at lower or higher copy numbers was also found. These kits did not achieve the desired limit of detection nor the desired specificity for the project at hand (Figs. 1, 2). The main reason behind the lack of sensitivity and low specificity might be due to the detection system used in these kits. The fluorescent probe used is often an intercalating dye, this is inherently problematic since any type of double stranded DNA present in solution will produce a non-specific fluorescent signal. Since the Bst 2.0 polymerase used in this amplification method does not possess 5’-3’ exonuclease activity it is not possible to use the hydrolysing probes normally used in qPCR.

Both kits showed positive amplification at ~ 11 min for high viral loads (Table 2) and positive controls, despite this, the sensitivity of the assay was not very good and did not allow for the robust and consistent detection of lower viral loads indicating that the amplification was still producing low amounts of amplified material and thus, there was room for improvement. It is clear that for moderate to high viral loads these kits can produce a significant response, despite this and most likely due to RT-LAMP using a combination of 6 different primers, achieving consistent results proved to be difficult.

**Table 2:** Time to positive amplification of respective genes using the different primer sets. Time to positive was calculated as being the sum of the average non-template control (NTC) RFU with 3 times the standard deviation.

Sample	Time to positive (min)
**N/E/As1e primer set**	
PC	14
0.1 cp/µL	–
0.5 cp/µL	–
1 cp/µL	–
10 cp/µL	17.5
100 cp/µL	15
**N/E primer set**	
PC	11
1 cp/µL	–
10 cp/µL	–
30 cp/µL	18
50 cp/µL	–
100 cp/µL	17
1 k cp/µL	12
**As1e primer set**	
PC	11
1 cp/µL	–
10 cp/µL	–
30 cp/µL	13
50 cp/µL	13
100 cp/µL	12
1 k cp/µL	11

Using a combination of the primer sets supplied with these kits it was possible to further increase the limit of detection of the system to 10 cp/µL of the stock solution (Fig. 1E,F). This was likely due to an increased probability of forming the high molecular weight double-stranded DNA products in RT-LAMP. Of note that even lower viral loads produced a fluorescent signal sporadically, this is indicative of low consistency and stochastic or on/off behaviour. We also observed this behaviour on the non-template control reactions (NTC). The multiplex approach increased the consistency of the results but still showed non-specific amplification within the desired reaction time, again, the main setback of this technology is using an intercalating dye for detection. From the turbidity of the reaction, it was possible to assess by the naked eye if the amplification was successful or not, thus there was always a way to infer if there was contamination present or not in the reaction (by comparing the turbidity of the NTC with that of the positive samples). Several reports showed that Guanidine hydrochloride (GndHCL) could be promising enhancers of both the RT-LAMP reaction and the Cas12a detection, we decided to investigate this compound^24,25^. By increasing the specificity of the primer—target sequence binding it is expected that the consistency of the results increases and that the stochastic term of the reaction stops being so relevant. This might improve our limit of detection (Fig. 2a). This enhancement by GndHCL is consistent with previously reported results and likely due to an enhanced base pairing mechanism or even modulation of enzyme activity to increase reaction speed and specificity. Also, supplementing this reaction with more Bst 2.0 polymerase (final concentration of 0.32 U/µL) on top of the one present in the master mix (undisclosed amount) greatly increased the speed of the re-action and even showed amplification at 1 copy/µL. These conditions reduced the start of amplification for higher viral load samples to 15 min while reducing the limit of detection to 1 copy/µL of stock solution and thus 0.2 copies in solution (Fig. 2b).

#### CRISPR/Cas12a based detection of synthetic RNA controls

**Figure 3:**
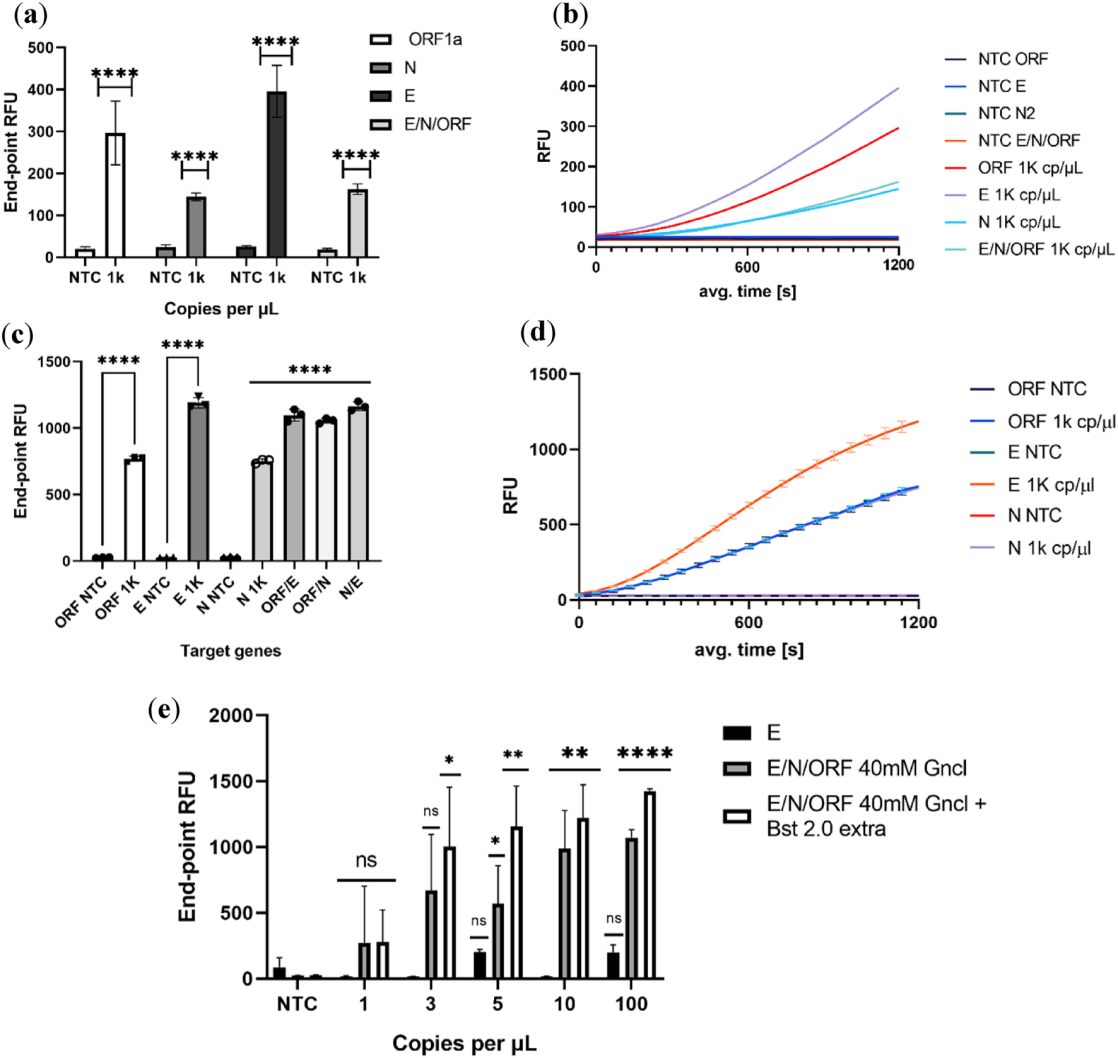
Detection of Sars-CoV-2 in the Cas12a reaction (2 step reaction). (**a**). End point fluorescence of the non-optimized reaction and the respective kinetics (**b**). (**c**) Detection of individual genes amplified by RT-LAMP by Cas12a using the optimized version and respective kinetic profile (**d**). (**e**) Limit of detection using a 3-primer set with guanidine hydrochloride and with extra Bst 2.0 polymerase. Statistical significance was determined by unpaired two-tailed t-test and all data were shown as mean ± S.D of 3 replicates. Asterisks indicate **P < 0.01; ***P < 0.001; ****P < 0.0001 and “ns” is non-significant.

When using the less optimized reaction as input to the Cas12a incubation step (2 step reaction), the end-point signal produced was very low (Fig. 3a) and no saturation plateau was observed (Fig. 3b). This was consistent with previously obtained results. Nonetheless, to maximize the end point signal some adjustments were made in the Cas12a reaction, namely, reducing incubation time at 37 °C from 30 to 20 min; using Diluent A to dilute the enzyme, adding the ssDNA probe after the incubation step and using 2 µL of input RT-LAMP reaction as it produces the best signal-to-noise ratio.

After all the reaction components were defined, the amplification of individual genes was once again tested (Fig. 3c) showing almost tenfold increase in the end point signal for the multiplex reaction and twofold higher for the individual reactions and an almost sigmoidal pattern for the enzymatic reaction (Fig. 3d). It is possible that the addition of the different components to the RT-LAMP reaction might enhance the CRISPR/Cas12a activity. The change of dilution buffer as well as the optimization of the best ratio of input reaction to enzyme reaction was critical. Higher amounts of RT-LAMP input showed to have an inhibitory effect on the Cas12a activity. Despite this, there was a synergistic effect between the optimization of the RT-LAMP reaction and the CRISPR/Cas12a detection (as expected) since the production of more amplicons and higher amount of DNA will inherently cause the higher detection rate and end point signal from the enzyme.

While the amplification of an individual gene (E) can yield a moderate positive signal at high viral loads, the combination of the multiple components makes the reaction more robust and with a final signal 5X higher than the original reaction (Fig. 3e). It was possible to observe a plateau for the enzyme kinetics (saturation) and increased reaction speed. This combination of multiple guide RNAs achieved lower or similar detection limits to tests that are currently on the market and thus can be a promising method to achieve cheap, point-of-care testing for the current and future outbreaks. The optimal reaction would cause the full consumption of the substrate and thus reach a plateau, the main goal was to try to achieve this reaction plateau for the lowest possible concentration of input RNA.

#### Melting curve analysis of amplified products

**Figure 4:**
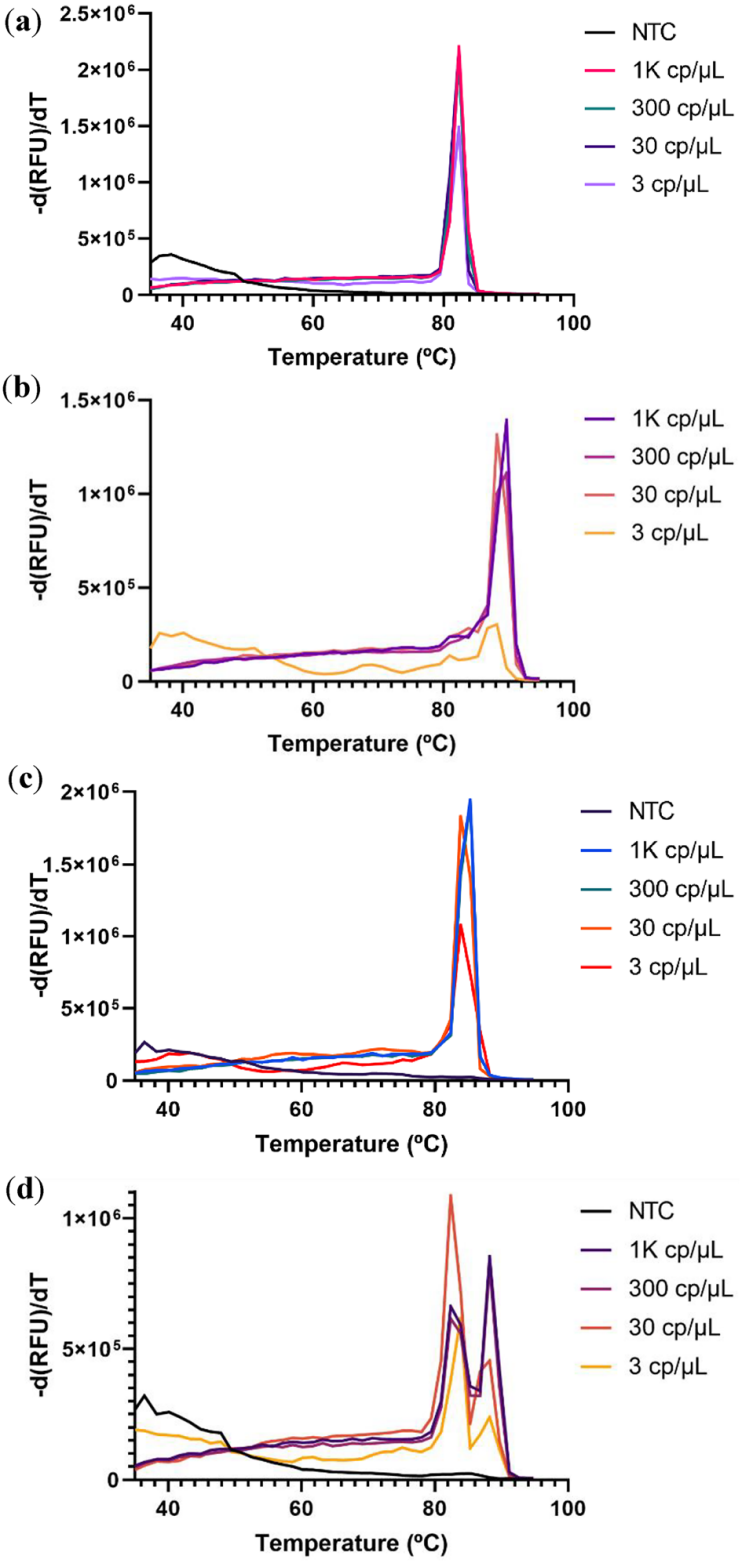
Melting curve analysis of the RT-LAMP reaction. Individual genes were amplified via RT-LAMP using different amounts of synthetic RNA resulting in the corresponding melting curve using E primers (**a**), N primers (**b**), As1 primers (**c**) and multiplex amplification (**d**). This analysis was used to validate the successful amplification in the RT-LAMP reaction using an intercalating dye from 35 to 95 °C with a heating ramp of 0.5 °C /s.

To verify that the amplification assay was indeed successful, we evaluated the fluorescence of the intercalating dye via melting curves (Fig. 4). As the temperature increases, the double strand of DNA produced via RT-LAMP was denatured and split into ssDNA Since we used an intercalating dye, as soon as dsDNA turns into ssDNA the fluorescence intensity dropped and could estimate of the strength of the hydrogen bonds of the fragment produced which is specific to the virus. From the obtained melting curves, we observed that the N gene presented non-specific amplification in the melting curve indicating a contamination of the non-template control wells (Fig. 4b). This contamination is always a concern with these isothermal amplification methods since there is exponential amplification rather than cycle based. Both individual genes, E gene in Fig. 4a and Orf1a in Fig. 4c, and multiplex (Fig. 4d) were amplified with specific melting points and without non-specific amplification. Clearly, lower copy amounts produced less amplicons for all the genes. In the multiplex situation it might be easier to detect lower copy amounts since there is a simultaneous amplification of different genes which can produce a favourable fluorescence signal when detected by Cas12a The specific melting points were 85.98 °C for the E gene, 90.34 °C for the N gene and 83.0 °C for the Orf1ab gene.

From the obtained results, it was possible to cross check the obtained results and have 100% confidence that both the RT-LAMP and the CRISPR/Cas12a reaction were giving accurate results. This served as the basis to assess the correct amplification of the individual genes (since the amplicons for each gene have a specific melting point) as well as a means to identify the relative proportion in which each gene is being amplified.

#### Limit of detection

**Figure 5:**
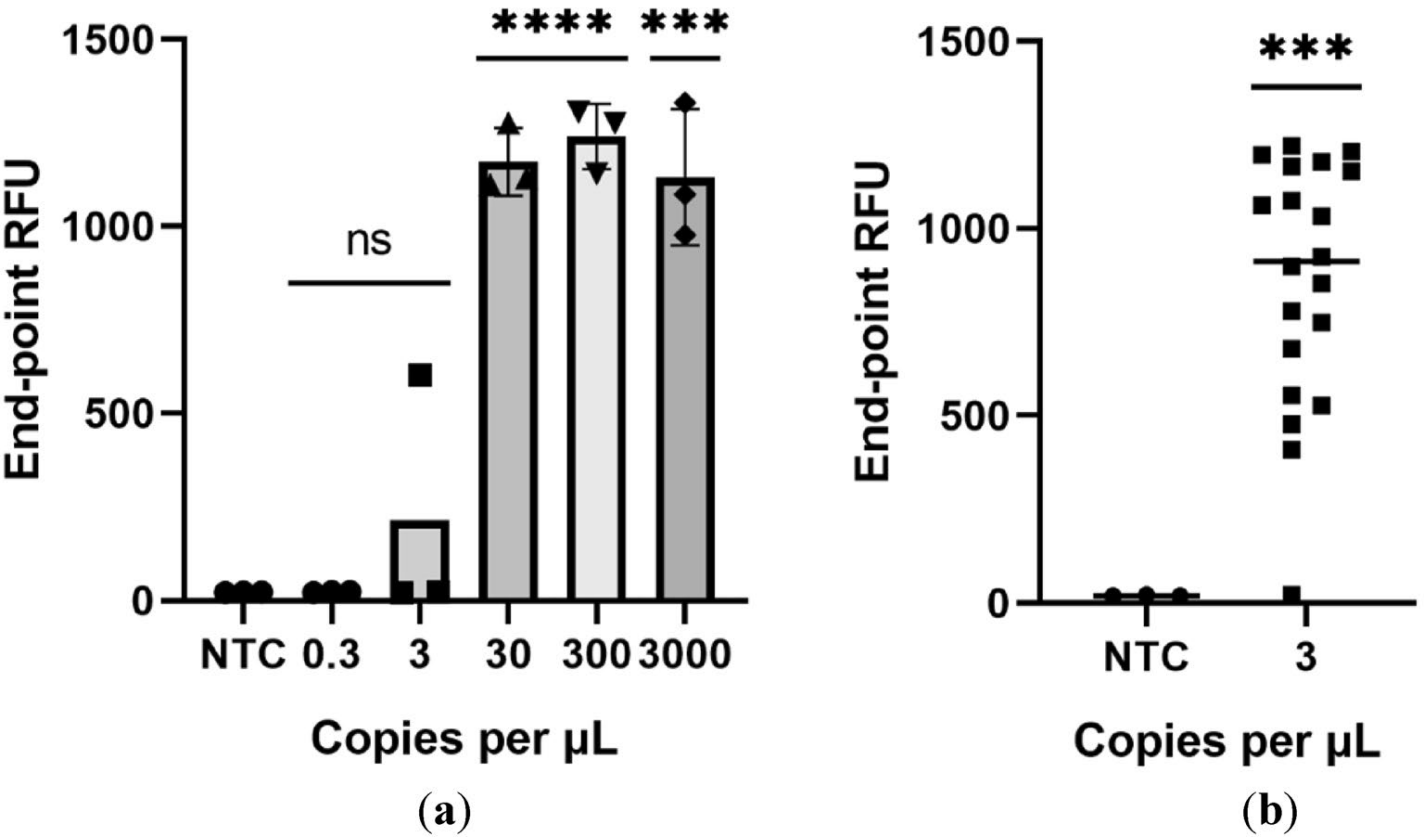
Limit of detection using the 2 step RT-LAMP/Cas12a reaction as recommended by the FDA. (**a**) End point detection of the different synthetic viral loads. (**b**) 20 replicates at this concentration revealed the detection of 19/20 indicating that this is the number of copies that is detected 95% of the time and thus our limit of detection. Statistical significance was determined by unpaired two-tailed t-test and all data were shown as mean ± S.D of 3 replicates. Asterisks indicate **P < 0.01; ***P < 0.001; ****P < 0.0001 and “ns” is non-significant.

After ensuring an amplification for all the genes, we assessed the limit of detection of the test using synthetic RNA controls in this 2-step system. The limit of detection proceeded as suggested by the FDA, namely, tenfold serial dilutions of synthetic RNA material were used (Fig. 5a), and 20 replicates of the hypothetical limit (19 out of 20 replicates detected, Fig. 5b). This brings our limit of detection to the 3–4 copy/µL range since all copy number above 3 produced 20 in 20 positive results (data not shown). Both higher copy numbers (4 copies/µL) and lower copy numbers (2 copies/µL) were tested to ensure this was the proper limit of detection, 4 copies/µL yielded 20/20 positive results while 2 copies/µL gave more than 1 negative in 20 samples.

#### Clinical validation

Having defined our detection limit using the synthetic control, the next step was to translate these results using clinical samples to have a more meaningful result about the performance of this testing method. Clinical samples were kindly provided by “Laboratório Nacional de Referência para o Vírus da Gripe e outros Vírus Respiratórios (LNRVG)” from “Instituto Nacional de Saúde Ricardo Jorge (INSA)”, Portugal. A total of 75 samples were tested where 25 were positive and 50 were negative, despite this our test manage to identify incorrectly defined samples as shown in Table 3. All positive samples were characterized with cycle threshold values using RT-qPCR (Table 4). In the RT-qPCR results, it is possible to observe that positive samples can sometimes amplify only 2 targets (or even 1 in some cases) out of the 3-target multiplex reaction. This can be problematic as the cycle threshold can vary from gene to gene, thus samples can be rendered negative if low abundance targets or stochastic effects lead to a negative amplification of some targets, while the others might be positive. Of utmost importance would be to use a system that can be fast while detecting several targets at the same time to avoid these problems. Hence a 3 gene multiplex system would provide more consistent and trustworthy results.

**Table 3:** COVsense testing method compared to the gold-standard technique.

	RT-qPCR result		Total
	Positive	Negative	
**KIT RT-LAMP COVSENSE**			
Positive	26 (TP)	0 (FP)	26
Negative	2 (FN)	47 (TN)	49
Total	28 (TP + FN)	47 (TN + FP)	75

**Table 4:** Comparison between positive clinical samples tested by RT-qPCR and our testing method. Cycle thresholds are represented under the specific amplified region ORF/N/E.COVsense testing method compared to the gold-standard technique.

Identification of sample	Sample type	Result PCR Fosun SARS CoV2	ORF	N	E	COVsense result
133999	Nasopharyngeal swab	Positive	11	10.03	12.6	Positive
134000		Positive	11.88	11	12	Positive
134402		Positive	10.46	9.04	11.14	Positive
134114		Positive	12.15	10.55	12.53	Positive
134118		Positive	25.73	25.9	26.24	Positive
134426		Positive	23.05	22.45	25.01	Positive
134482		Positive	27.22	25.44	26.47	Positive
134504		Positive	28.19	27.07	27.49	Positive
134519		Positive	23.55	22.31	22.73	Positive
134522		Positive	12.62	11.35	12.25	Positive
134579		Positive	15.19	14.33	16.16	Positive
134584		Positive	17	15.08	17.08	Positive
134627		Positive	13.09	11	14	Positive
134609		Positive	32.41	32.06	34.06	Positive
134776		Positive	30.14	24.9		Positive
134916		Positive	14.36	16.19	15.14	Positive
134917		Positive	24.51	24.4	25.14	Positive
134918		Positive	37.79	29.32	32	Negative
134919		Positive	13.15	12.53	14.06	Positive
134920		Positive	18.34	17.84	15.19	Positive
134921		Positive	20.61	19.62	21.12	Positive
134922		Positive	20.85	16.5	19.16	Positive
134924		Positive	26.11	24.51	26.05	Positive
134925		Positive		31.1	35.36	Negative
134926		Positive	25.26	24.03	25.37	Positive

Samples ranging from Ct 9 to a Ct of 37 were tested with excellent overall agreement and specificity for Ct below 35. Despite this, the sensitivity of this test is almost at the threshold of the gold-standard considering that samples are considered negative for the virus with a threshold above 37. These results provide our testing method with 100% of specificity since there were no false positive results among the negative samples that were tested, and 93% of positive agreement with a total agreement of 97% (Table 5). Rnase P tests were not made (using the CRISPR/Cas12a reaction, these were analysed via RT-qPCR) due to low sample volume. Despite this, Rnase P detection was validated in our system (Supplementary Figure A4).

**Table 5:** Agreement between the RT-qPCR test and the COVsense RT-LAMP CRISPR/Cas12a test.

Specificity of COVsense compared to RT-qPCR		
Positive percent agreement (%)	TP/(TP + FN) × 100%	93%
Negative percent agreement (%)	TN/(TN + FP) × 100%	100%
Total agreement PA (%)	(TP + TN)/(TP + TN + FN + FP) × 100%	97%
Theoretical agreement Pe	[(TP + FP) (TP + FN) + (FN + TN) (FP + TN)]/(TP + TN + FN + FP)2	0,54
Coefficient K	(PA − Pe)/(1 − Pe)	0,94

**Figure 6:**
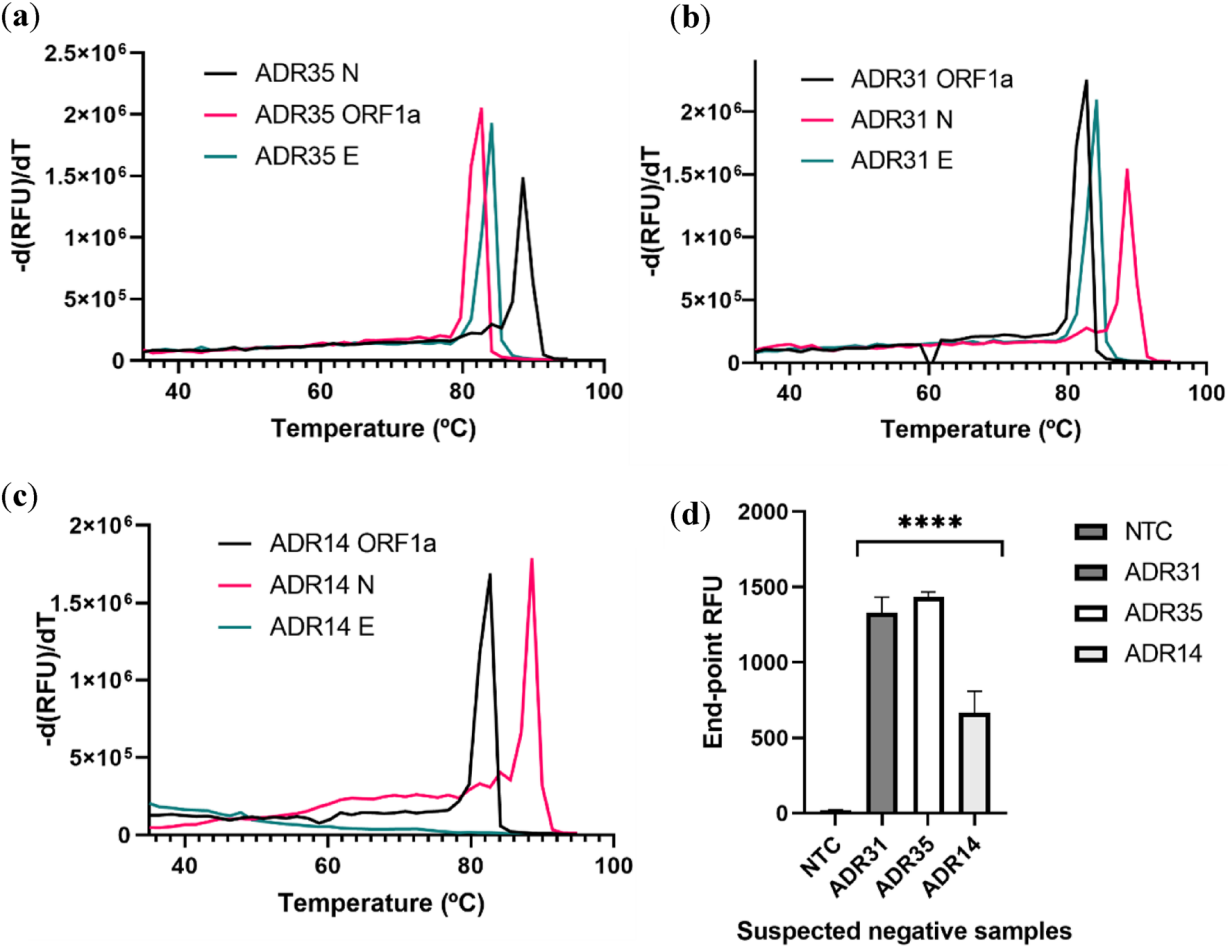
Melting curve analysis from the RT-LAMP reaction of clinical samples. Samples that were considered SARS-CoV-2 positive by INSA were further analysed. Melting curve for sample ADR35 (**a**), ADR31 (**b**) and ADR14 (**c**) using the primers sets for the 3 different genes. (**d**) End-point fluorescent signal measured in the Cas12a reaction. Statistical significance was determined by unpaired two-tailed t-test and all data were shown as mean ± S.D of 3 replicates. Asterisks indicate **P < 0.01; ***P < 0.001; ****P < 0.0001 and “ns” is non-significant.

After testing the negative samples that were provided, we verified that three (ADR14, ADR31, and ADR35) negative samples tested positive, two of which were positive for other respiratory viruses. After re-testing the samples both by RT-qPCR and COVsense we confirmed that these were indeed positive samples (Table 6) that were detected both by our method and by the melting curve analysis using RT-LAMP (Fig. 6a–c) and by the Cas12a reaction (Fig. 6d).

**Table 6:** Positive samples that were previously categorized as negative with diverse cycle threshold values were re-tested by RT-qPCR.

Identification of sample	Sample type	Other respiratory viruses	PCR Fosun SARS CoV 2	ORF	N	E	COVsense result
ADR14	Nasopharyngeal swab	RSV A	Positive	–	32.5	34.85	Positive
ADR31		Rhinovirus	Positive	27.54	24.8	25.64	Positive
ADR35		Negative	Positive	15.93	13.7	15.33	Positive

The samples were poorly processed (probably due to being positive to other respiratory viruses) and were mislabelled and detected by our system. The RT-qPCR characterization showed samples with high Cts meaning that the most likely cause for negative results in the high Ct samples were probably due to degradation. Further tests using internal controls (Rnase P) were made for samples that tested negative and the results were negative (data not shown). These tests should indeed have been made in parallel and not in such a different time window, Rnase P tests should have also been processed at the same time to determine sample degradation at the time of detection. Despite this, it is clear that the detection sensitivity and specificity of this testing method could greatly simply the workload of laboratory staff when it comes to labelling high volumes of samples.

#### Detection of synthetic controls in saliva samples

**Figure 7:**
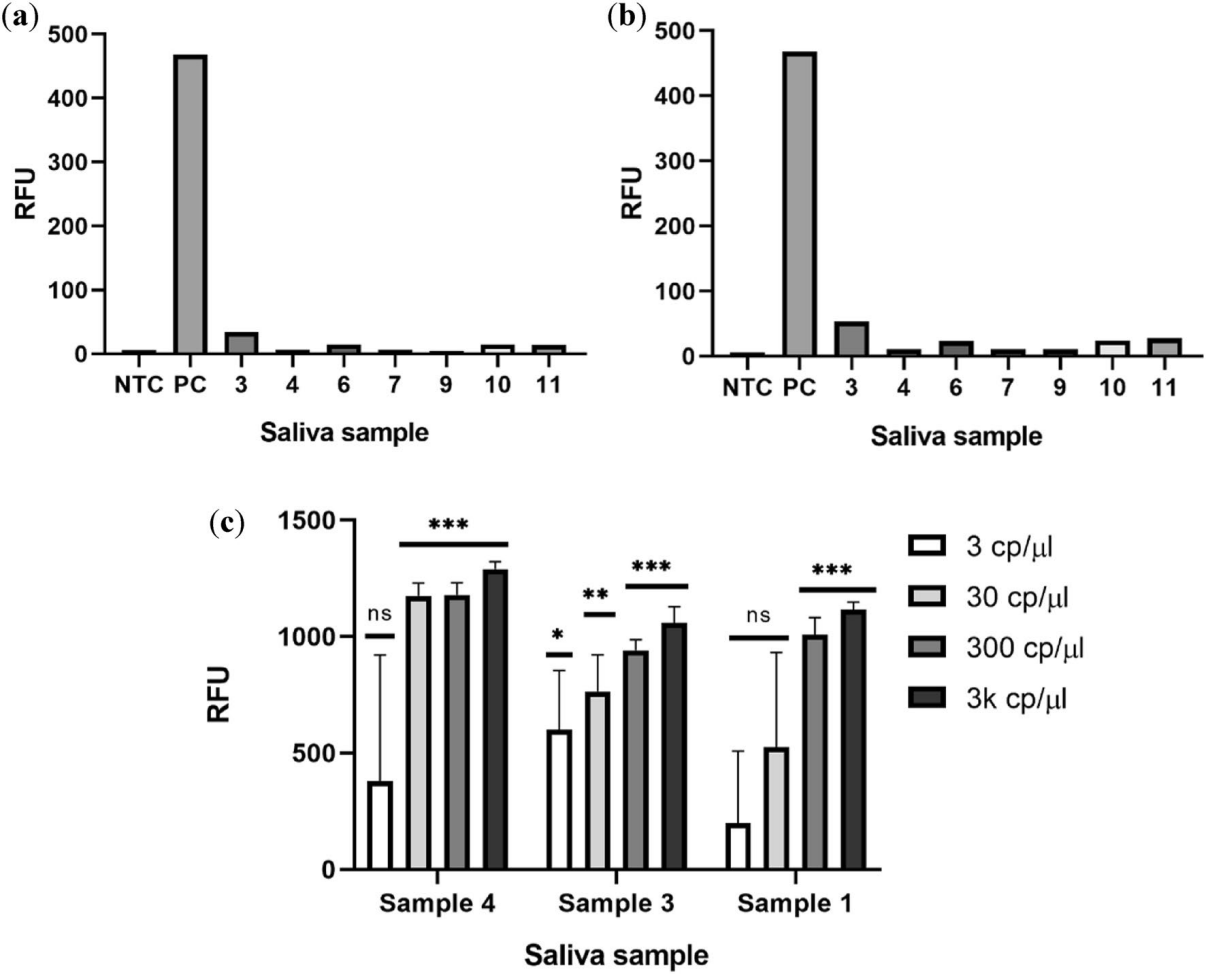
Inactivation of nucleases in saliva samples and limit of detection of spiked samples. RnaseAlert substrate nuclease detection system reaction for the different saliva samples using the SHINE buffer (**a**) and the Tween-20 buffer (**b**) in a 1:1 proportion. (**c**) End-point fluorescence signal in the Cas12a-mixture of spiked saliva samples processed using the SHINE buffer. Statistical significance was determined by unpaired two-tailed t-test and all data were shown as mean ± S.D of 3 replicates. Asterisks indicate **P < 0.01; ***P < 0.001; ****P < 0.0001 and “ns” is non-significant.

We proceeded to test our assay in saliva samples. Two different buffers were tested for the inactivation and removal on inhibitors from saliva. The two buffers tested have slightly different compositions, the first one derives from the SHINE protocol (Fig. 7a) and is composed of 100 mM TCEP and 1 mM EDTA, pH = 8 and the second one is composed of 2.5 mM TCEP, 1 mM EDTA and 0.1% Tween-20 (Fig. 7b). Each saliva sample was heat inactivated by incubating at 65 °C for 30 min. A 2 × solution of each of the buffers was mixed in a 1:1 proportion with saliva samples. These buffers were heat treated for 5 min at 95 °C and 22.5 µL of each was used to check for the presence of nucleases by incubating with the RnaseAlert substrate nuclease detection system (IDT), this takes advantage of a fluorescent RNA substrate in each reaction that will produce a signal if there are any Rnases in solution. In the positive control (PC) well, Rnase A is added. These solutions were incubated for 1 h at 37 °C in a 384-well plate. It was possible to observe that there was no need to use Rnase Inhibitor when using these experimental conditions.

Saliva samples that were previously spiked with known amounts of inactivated virus and/or total swab controls were amplified via RT-LAMP and 2 µL of this reaction was used as input for 20 µL of the RNP mixture (2-step reaction). The end point signal for the different concentrations is shown in Fig. 7c. Of note that the NTC reactions remained negative after 24 h.

It was possible to achieve a limit of detection below 300 cp/µL for sample 1 (which was blind tested) while for other samples the limit was of 30 cp/µL (10X LOD for the extracted genetic material). There is the possibility that contaminants were present in sample 1, namely, poor accumulation of gargle leading to sample degradation, drinking coffee or eating before the 30 min asked to provide the sample.

**Figure 8:**
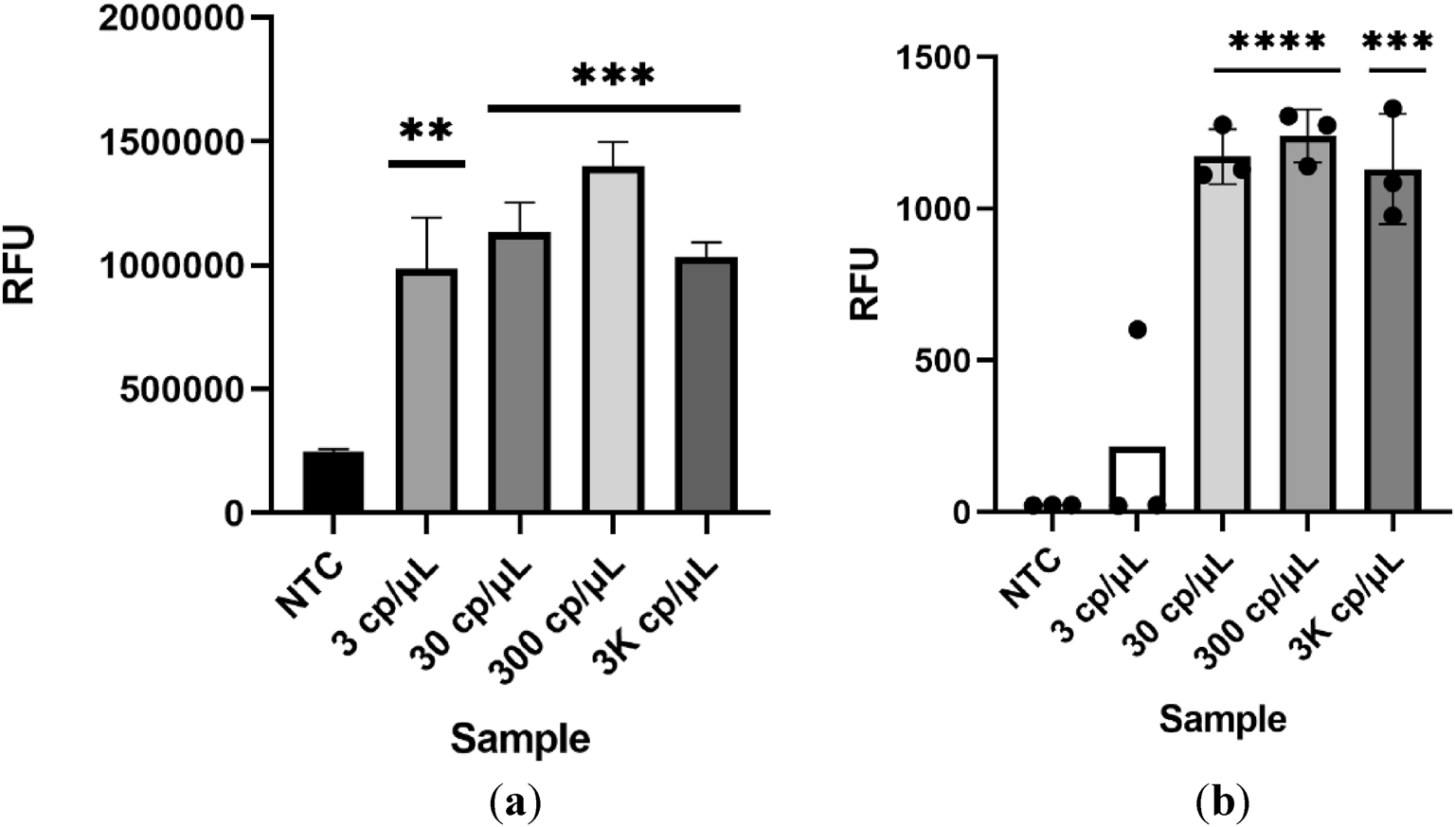
One-pot RT-LAMP-CRISPR/Cas12a detection of synthetic RNA. End point RFU signal using a dedicated equipment (**a**) after 10 min and Varioskan Lux multimode plate reader. (**b**) Statistical significance was determined by unpaired two-tailed t-test and all data were shown as mean ± S.D of 3 replicates. Asterisks indicate **P < 0.01; ***P < 0.001; ****P < 0.0001 and “ns” is non-significant.

In order to reduce contamination risks of the reaction vessel, workstation and equipment, there as the need to have a one-pot reaction. This hypothesis was put to the test, after optimization (data not shown) the best ratio was found to be 50 µL of Cas12a solution to 20 µL of RT-LAMP reaction. After 30 min of reaction time at 63 °C, the Eppendorf strip was briefly centrifuged (spin down) to mix the enzymatic solution located in the lid of the Eppendorf with the RT-LAMP reaction. This mixture was incubated at 37 °C for 10 min (Fig. 8).

The end point signal was found to have high significance after 20 min using a dedicated device (Fig. 8a) comparable to the results obtained using a very sensitive microplate reader (Fig. 8b) also, no unspecific signal was detected even after 24 h (Data not shown). In this case, detection of 3 copies per microliter of viral RNA also showed a on/off behaviour being detected sporadically, despite this, when this low viral load was detected, the result showed high significance. These results provided the indication that it was possible to achieve the same detection limit as the 2-step method with high significance and no non-specific signal even after 24 h. More tests are needed to compare the performance of this method with the 2-step version. The most important factor to consider is the condition in which the saliva sample is collected. This is of major importance since there are many inhibitory compounds present in saliva, the ingestion of certain kinds of food/beverages before the 30 min required for sample collection will also greatly impair the assay.

Using the dedicated equipment, it is possible to process samples and determine the result needing only a centrifuge to mix the RNP reaction with the RT-LAMP amplification. Thus, in a short period of time we were able to create a proof-of-concept one pot RT-LAMP CRISPR/Cas12a molecular test that will be integrated inside a fully automated device. This test uses large volumes (70 µL), lower costs due to lower enzyme and guide RNA concentration, surpasses the current reagent and consumable shortage that was caused by the actual pandemic while still retaining a comparable limit of detection to the gold-standard RT-qPCR. The only external equipment required to perform this kind of testing is a heat source (from 37 to 65 °C) a light source at 485 nm and a spectrophotometer or a light sensor to detect the fluorescence signal. This testing method shows great potential to integrate the next generation of molecular diagnostics overtaking PCR as the gold standard to enable highly sensitive point-of-care technologies.

## Conclusions

While RT-PCR has high turnaround times and requires experienced or specialized personnel to be performed, expensive equipment, and suitable facilities, isothermal amplification methods provide for point-of-care testing while retaining higher sensitivity than antigen tests in a low-resource environment. By simultaneously detecting 3 genes of the virus (E/N/Orf1a) the probability of false positives is greatly reduced (achieving 100% specificity). The low concentration of reagents used also provides a cheaper alternative to currently available methods as well as the combination with open-source reagents for an RNA extraction-free process making it suitable for point-of-care testing. This innovative testing method poses several advantages when compared to other tests that are already commercialized, this is a one-pot testing method, there is no need to open any type of container minimizing the probability of having contaminations in the workspace; completely erases the need to use a thermocycler, highly versatile; and proved to be very sensitive (3 copies/µL) with a total assay time of 40 min while detecting 3 different genes simultaneously leading to a higher accuracy, response time as well as consistency.

Commercially available RT-LAMP kits provide reasonable detection of SARS-CoV-2 but without the needed robustness or specificity. We successfully implemented different reaction additives that had been previously described onto our assay (Guanidine HCL) allowing for a more consistent and robust response. From the data presented, multiplexing the RT-LAMP reaction yields higher signal to noise ratio, higher specificity and higher sensitivity. We can observe that the consistency of the readout increased for lower copy amounts although at the cost of a small decrease in the reaction speed. When comparing with the single plex or duplex reaction, there is less variability in the obtained results. The synergy between the assembled reaction components allowed for an improved Cas12a reaction, this provided a tenfold increase in the end point RFU signal when comparing with the initial single plex reaction where the detection of a single gene provided the best signal to noise ratio.

By analysing the single plex melting curves of the RT-LAMP reaction we can get some insight on why this happens, it is clear that lower viral copy amounts yield lower DNA amplification and thus will be more difficult to detect. Despite this, In the multiplex case, we can observe the different melting points for the different genes, corresponding to a successful amplification of the target genes meaning that we can broaden the range of detection of this testing method. Even for lower viral copy amounts, a sample that would otherwise be labelled as negative (due to the detection of a target gene that was present in low amounts) can be correctly identified since there is a lower probability of all 3 targets not being amplified. Therefore, combining multiplex RT-LAMP amplification of 3 different genes of the SARS-CoV-2 virus with the simultaneous detection of these genes by CRISPR/Cas12a it was possible to achieve very high sensitivity while retaining 100% specificity thus eliminating the major problem of isothermal amplification methods which is non-specific amplification, while tackling the RT-qPCR drawback of incorrect sample characterization due to failure of amplification of 1 or more genes. The detection of synthetic RNA controls in saliva samples poses a great step forward in a less intrusive testing method that can be used more recurrently. This can have major implications on new diagnostic technologies since it is possible to reduce time of amplification without sacrificing specificity and bring testing capabilities to areas where they were not accessible before. The universal character of this testing method proves to have endless possibilities when it comes to detecting viral infection, bacteria, or other types of pathogens since the adaptation process of this method only requires changing primers and guide RNAs for different targets.

## Materials and methods

### Nucleic acid preparation

SARS-CoV-2 target sequences were designed using the available genomic sequence from NCBI as of 30 January 2021. These sequences were then aligned with bat SARS-like CoV and MERS to verify cross-reactivity. LAMP primers were based on previously published sequences. Considering currently used target sites by the CDC and WHO, gRNAs were designed for the E gene, N gene, and the ORF1ab as well as a sample control gRNA that that was designed for previously published RNase P POP7 RT-LAMP primers. These were either based on open-source sequences (gRNA for E and N and RNase P) that had already been published or designed by root (Orf1ab gRNA). For the in vitro testing a synthetic ssRNA control was used. Oligonucleotide DNA sequences were synthesized by IDT with a yield of 25 nmole using standard desalting and then diluted to 100 µM of final concentration in nuclease-free water (IDT). The reporter molecule was an oligonucleotide functionalized with FAM (5´-end) and with BHQ (3’-end) using HPLC purification with a yield of 1µmole based on the sequence used by DETECTR. gRNAs were ordered from IDT with a yield of 2 nmol and standard desalting all with 5’ and 3’ Alt-R end-blocking modifications and standard desalting with a stock solution of 50 µM.

### 5X primer mixture with guanidine

LAMP primer mixtures (1X final, 10X stock diluted with guanidine to 5X) were assembled using a final concentration of 0.2 µM of F3/B3, 0.4 µM of LF/LB, and 1.6 µM of FIP/BIP in nuclease-free water. The stock solution of each primer (N/E/ORF1ab) 10X was mixed in equimolar proportions with guanidine (200 mM in 5X primers mix and 40 mM final concentration) to a concentration of 5X and this was the solution that was used for the simultaneous detection of the 3 different genes.

### RT-LAMP fluorescent assays

For the detection of SARS-CoV-2 RNA, several precautions were used to prevent sample contamination, such as using nuclease decontamination solution or RNase Away, filtered tips, and nuclease-free certified reagents in a dedicated clean bench as well as a laminar flow chamber with 20 min of UV decontamination when opening the plates was needed. For the RT-LAMP Vienna biocentre 12,13 reactions 11 µL of tube 2A-LAMP MIX was mixed with 2.5 µL of tube 3A-SARS-CoV-2 As1 primer and with 2.5 µL of tube 4A-HNB dye and where referred, 0.4 µL of tube 7A-FLUO DYE (SYTO-9). Of this mixture, 16 µL were dispensed per well and 4 µL of extracted RNA was added, vortexed, spined down, and incubated for 35 min at 63 °C. For the RT-LAMP mixture by SARS-CoV-2 Rapid Colorimetric LAMP Detection Assay Protocol (NEB #E2019) 12.5 µL of WarmStart Colorimetric LAMP 2X Master Mix with UDG, 2.5 µL of SARS-CoV-2 LAMP Primer Mix (N/E), 2.5 µL Guanidine Hydrochloride (40 mM of final concentration), 7.5 µL of nuclease-free-water and 2.0 µL of either sample nucleic acid (Extracted RNA), SARS-CoV-2 Positive Control (N gene) to a total reaction volume of 25 µL. When using WarmStart® LAMP Kit (DNA & RNA), for a final volume of 20.4 µL the reaction was assembled with 10 µL of WarmStart® Colorimetric LAMP 2X Master Mix (NEB #M1804), 4 µL of an equimolar 5X Primer mix (N/E/Orf1ab) with 200 mM of Guanidine HCL, 0.7 mM of dUTP, 0.7 mM of MgSO_4_, 0.02 U/µL of UDG, 0.32 U/µL of Bst 2.0 WarmStart® DNA Polymerase (#M0538M), 0.4 µL of 50X LAMP dye and 0.92 µL of nuclease-free-water, to this mixture 4 µL of extracted RNA samples was added in different concentrations. These reactions were incubated at either 65 °C or 63 °C where specified for 30 min. For the real-time fluorescent detection of the LAMP result, a 384-well plate was used in QuantStudio™ 5 Real-Time PCR Instrument using 30 cycles of 1 min at the specified temperature using the standard FAM emission and excitation filters. End-point measurements for the RT-LAMP reaction (Fig. 1B,D,F) where done using Varioskan™ LUX multimode microplate reader. Melt curve analysis was performed from 35 to 95 °C with a heating ramp of 0.5 °C/s.

### CRISPR assay

For the CRISPR-based detection system, We used Lba Cas12a from New England Biolabs (EnGen® Lba Cas12a (Cpf1) #M0653T). Firstly, Cas12-gRNA complexes were generated by pre-incubating LbaCas12a (50 nM, final concentration) with the gRNA for each gene (50 nM final concentration, the gRNA targeting each gene—E/N/ORF1a—was added in equal proportion)) for 20 min at 37 °C in 1X NEBuffer 2.1 for a final volume of 20 µL. After the incubation, the fluorescent reporter molecule was added at a final concentration of 4 µM and placed on ice. After the desired target amplification by RT-LAMP, 2 µL of each reaction is added to 20 µL of the Cas12-gRNA mixture and incubated at 37 °C for 20 min. This RNP detects amplicons produced by the RT-LAMP reaction simultaneously for all the 3 different genes (E/N/ORF1ab). The real-time fluorescence acquisition was done using the Varioskan™ LUX multimode microplate reader using 20 cycles of 1 min. One pot RT-LAMP/Cas12a reactions were tested using 50 µL of Cas12a solution to 20 µL of RT-LAMP reaction. After 30 min of reaction time at 63 °C, the Eppendorf strip was briefly centrifuged (spin down) to mix the enzymatic solution located in the lid of the Eppendorf with the RT-LAMP reaction. This mixture was incubated at 37 °C for 10 min.

### In silico analysis

All SARS-CoV-2 sequences were aligned using Clustal omega using available genomes from GenBank (NCBI) as of January 2021. The specific target sites by LbaCas12a were compared with human coronavirus genomes (NC_045512.2), bat SARS-like CoV (NC_014470.1), and with MERS (NC_019843.3) to assess cross-reactivity. The LAMP primers were analysed via PrimerExplorer v.5 (https://primerexplorer.jp/e/) or based on previously published primers. All designed gRNAs were 100% specific (Figs. A1–A3) with Expect (E) values of 5 × 10^–6^ in the plus/plus strand, the E gene gRNA enables the detection of SARS-CoV-2 (100%) bat coronavirus (95.24%) and MERS (93.75%). All RT-LAMP primers have been previously validated as highly specific with minimal cross reactivity as confirmed by the in-silico analysis.

### Human clinical sample collection and preparation

Clinical nasopharyngeal swab samples were kindly provided by Instituto Ricardo Jorge (LNRVG) either from patients infected with SARS-CoV-2 (or other respiratory viruses) or from RT-qPCR negative individuals. Sample RNA was extracted according to the manufacturer's protocol.

### Real-time RT-qPCR assay

The determination of positive or negative samples was confirmed by RT-PCR using “Novel Coronavirus (2019-nCoV) RT-qPCR Detection Kit (Fosun 2019-nCoV qPCR), this kit sets the maximum cycle threshold at 36 for a positive signal. This assay was performed on the Biorad Real-Time PCR system instrument.

### Analytical validation (LoD)

LoD is the lowest detectable concentration at which around 95% of all true positive replicates test positive. The FDA recommends testing a dilution series of three replicates per concentration with inactivated controls and then confirm the final concentration with 20 replicates. The analytical validation of the detection kit was performed by using synthetic RNA controls provided by Twist Biosciences. From this stock, ten-fold serial dilutions were made until the detection limit was reached (2/3 true positives were detected). This limit was then tested in 20 replicates achieving a total of 19/20 for the initial sample solution of 3 copies/µL.

### Clinical validation

The analysis was carried out on 75 anonymized nasopharyngeal swabs with negative (n = 50) and positive (n = 25) results by RT-qPCR provided by INSA. The samples were freshly extracted on the 23rd of August 2021 and remained stored in the laboratory until they were delivered to our team on the 24th of August 2021. Once received at − 20 °C, the 75 samples were analysed for the E, Orf1ab, N viral genes as well as internal control such as Rnase P, to determine sensitivity and specificity values, as well as repeatability and limit of detection (analytical sensitivity). All experimental protocols were approved by Instituto Ricardo Jorge, Laboratório Nacional de Referência para o Vírus da Gripe (LNRVG). All methods were carried out in accordance with relevant guidelines and regulations.

### Processing of saliva samples

Saliva samples were retrieved from healthy, covid negative donors and kept at − 20 °C. Each saliva sample was heat inactivated by incubating at 65 °C for 30 min. Two different buffers were tested, the first one is composed of 100 mM TCEP and 1 mM EDTA, pH 8 and the second one is composed of 2.5 mM TCEP, 1 mM EDTA and 0.1% Tween-20. A 2 × solution of each of the buffers was mixed in a 1:1 proportion with saliva samples that were previously spiked with known amounts of in-activated virus and/or total swab controls. These were heat treated for 5 min at 95 °C and 22.5 µL of each was used to check for the presence of nucleases by incubating with the RnaseAlert substrate nuclease detection system (IDT), this takes advantage of a fluorescent RNA substrate in each re-action that will produce a signal if there are any Rnases in solution. In the positive control (PC) well, Rnase A is added. These solutions were incubated for 1 h at 37 °C in a 384-well plate. For the detection tests, these samples were spiked with SARS-CoV-2 RNA controls from Twist Biosciences and treated using the developed protocol (1:1 in 2X Buffer with a final concentration of 100 mM TCEP and 1 mM EDTA pH = 8 for 5 min at 95 °C). The processed samples were then spiked with different initial concentrations. Informed consent was obtained from all subjects and/or their legal guardian(s).

### Statistical analysis

Data were analysed using GraphPad Prism version 8.0.0 for Windows, GraphPad Software, San Diego, California USA, www.graphpad.com. Statistical significance was determined by unpaired two-tailed t-test and all data were shown as mean ± S.D of 3 replicates. Asterisks indicate **P < 0.01; ***P < 0.001; ****P < 0.0001 and “ns” is non-significant.

## Supplementary Information

Supplementary Information.

## Data Availability

The datasets used and/or analysed during the current study available from the corresponding author on reasonable request.
