# Supplementary material for: Rapid, multiplex detection of SARS-CoV-2 using isothermal amplification coupled with CRISPR-Cas12a
Source: Sci Rep. 2023 Jan 16;13:849. doi: 10.1038/s41598-022-27133-7 (PMC9842216; doi:10.1038/s41598-022-27133-7)
Supplement: Supplementary file 1 — Supplementary Information. [file 41598_2022_27133_MOESM1_ESM.docx]

**Supplementary information file for Rapid, multiplex detection of SARS-CoV-2 using isothermal amplification coupled with CRISPR-Cas12a**

**Appendix A**

**Table A1 -** Fluorescent reporters and guide RNAs used in this study.

| **Name** | **Sequence (5’-3’)** |
| --- | --- |
| FAM Reporter 1 | 5'/56-FAM/TTATTATT/3BHQ1/3' |
| FAM Reporter 2 | 5'/56-FAM/TTATT/BHQ/-3' |
| RNaseP gRNA | UAAUUUCUACUAAGUGUAGAUAAUUACUUGGGUGUGACCCU |
| gRNA ORF1 V1 | UAAUUUCUACUAAGUGUAGAUUGUGCUGACUCUAUCAUUAU |
| gRNA ORF 1 V2 | UAAUUUCUACUAAGUGUAGAUUCACGCACUCAAAGGGAUUG |
| gRNA N | UAAUUUCUACUAAGUGUAGAUCCCCCAGCGCUUCAGCGUUC |
| gRNA E | UAAUUUCUACUAAGUGUAGAUGUGGUAUUCUUGCUAGUUAC |

**Table A2** - Oligonucleotide sequences used in this study.

| **Primer Set** | | **Sequence (5'-3')** |
| --- | --- | --- |
| **As1** [19] | |  |
| F3 | | CGGTGGACAAATTGTCAC |
| B3 | | CTTCTCTGGATTTAACACACTT |
| FIP | | TCAGCACACAAAGCCAAAAATTTATCTGTGCAAAGGAAATTAAGGAG |
| BIP | | TATTGGTGGAGCTAAACTTAAAGCCCTGTACAATCCCTTTGAGTG |
| LF | | TTACAAGCTTAAAGAATGTCTGAACACT |
| LB | | TTGAATTTAGGTGAAACATTTGTCACG |
| **E1 gene** [17] |  | |
| F3 | TGAGTACGAACTTATGTACTCAT | |
| B3 | TTCAGATTTTTAACACGAGAGT | |
| FIP | ACCACGAAAGCAAGAAAAAGAAGTTCGTTTCGGAAGAGACAG | |
| BIP | TTGCTAGTTACACTAGCCATCCTTAGGTTTTACAAGACTCACGT | |
| LF | CGCTATTAACTATTAACG | |
| LB | GCGCTTCGATTGTGTGCGT | |
| **E2 gene** [10] |  | |
| F3 | CCGACGACGACTACTAGC | |
| B3 | AGAGTAAACGTAAAAAGAAGGTT | |
| BIP | ACCTGTCTCTTCCGAAACGAATTTGTAAGCACAAGCTGATG | |
| FIP | CTAGCCATCCTTACTGCGCTACTCACGTTAACAATATTGCA | |
| LF | TCGATTGTGTGCGTACTGC | |
| LB | TGAGTACATAAGTTCGTAC | |
| **N1 gene** [17] |  | |
| F3 | ACCAGGAACTAATCAGACAAG | |
| B3 | GACTTGATCTTTGAAATTTGGATCT | |
| FIP | TTCCGAAGAACGCTGAAGCGGAACTGATTACAAACATTGGCC | |
| BIP | CGCATTGGCATGGAAGTCACAATTTGATGGCACCTGTGTA | |
| LF | GGGGGCAAATTGTGCAATTTG | |
| LB | CTTCGGGAACGTGGTTGACC | |
| **N2 gene** [10] |  | |
| F3 | AACACAAGCTTTCGGCAG | |
| B3 | GAAATTTGGATCTTTGTCATCC | |
| FIP | TGCGGCCAATGTTTGTAATCAGCCAAGGAAATTTTGGGGAC | |
| BIP | CGCATTGGCATGGAAGTCACTTTGATGGCACCTGTGTAG | |
| LF | TTCCTTGTCTGATTAGTTC | |


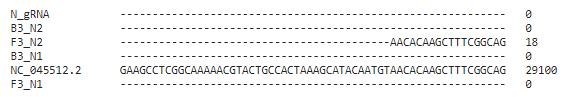

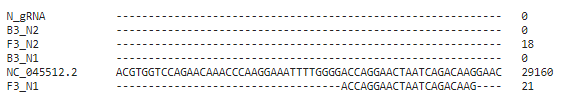

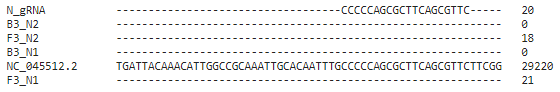

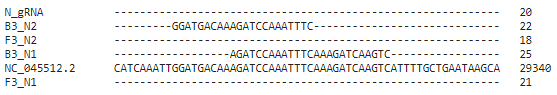


**Figure A1** - Alignment of the SARS-CoV-2 genome using Clustal omega with the respective primers and guide RNA target sequences for the N gene.


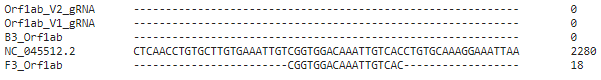

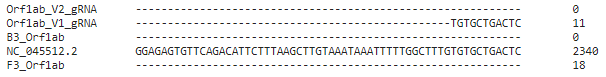

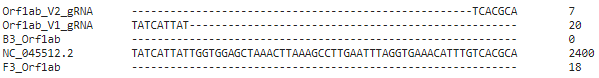

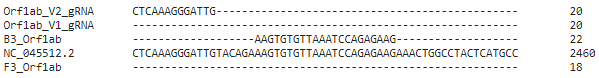


**Figure A2** - Alignment of the SARS-CoV-2 genome using Clustal omega with the respective primers and guide RNA target sequences for the Orf1ab gene.


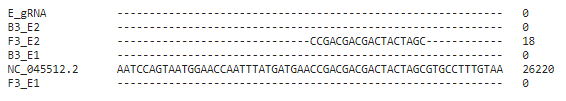

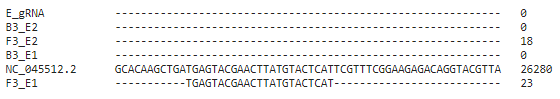

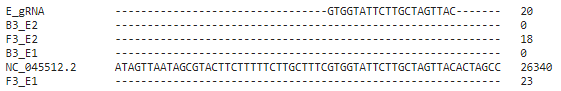

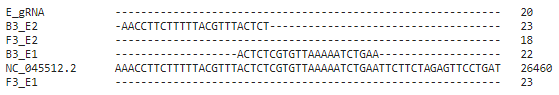


**Figure A3** - Alignment of the SARS-CoV-2 genome using Clustal omega with the respective primers and guide RNA target sequences for the E1 gene.

We could observe amplification in all genes (except E gene for ADR14). The positive samples, on the other hand, tested negative due to low viral loads (high Ct values) and could not be detected by our system, sample CV134925 on the other hand tested positive (Figure A4a) in the melt curve analysis but for very low amounts of viral material (N gene, Figure A4b) which is in accordance with the viral load detected by RT-qPCR (very high Ct values.

For a sample to be correctly validated as positive or negative an extra step is necessary. An internal control is often used to verify that the extraction procedure of the sample was done correctly. By using Rnase P as an internal control (Figure A4c) our system can have the maximum confidence in the produced results with high significance (Figure A4d). This proves to be a proof-of-concept work to develop a fully functional RT-LAMP/Cas12a diagnostic test.

(**b**)

(**a**)


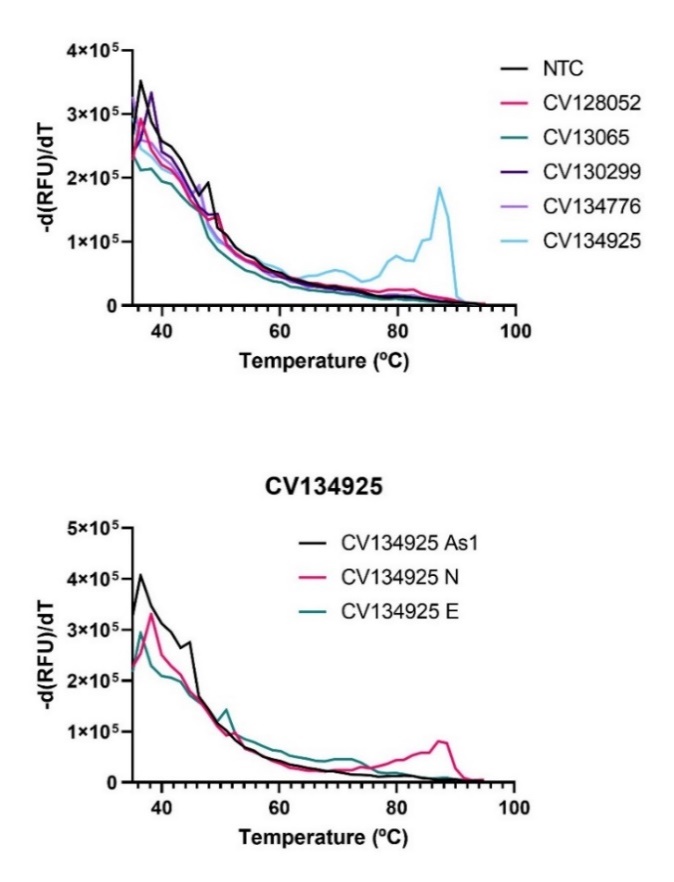

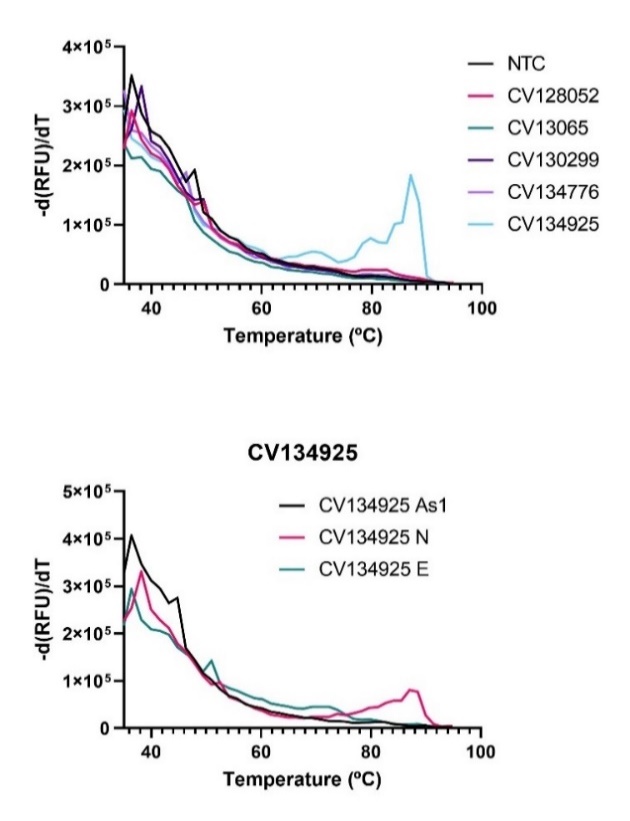


(**c**)

(**d**)


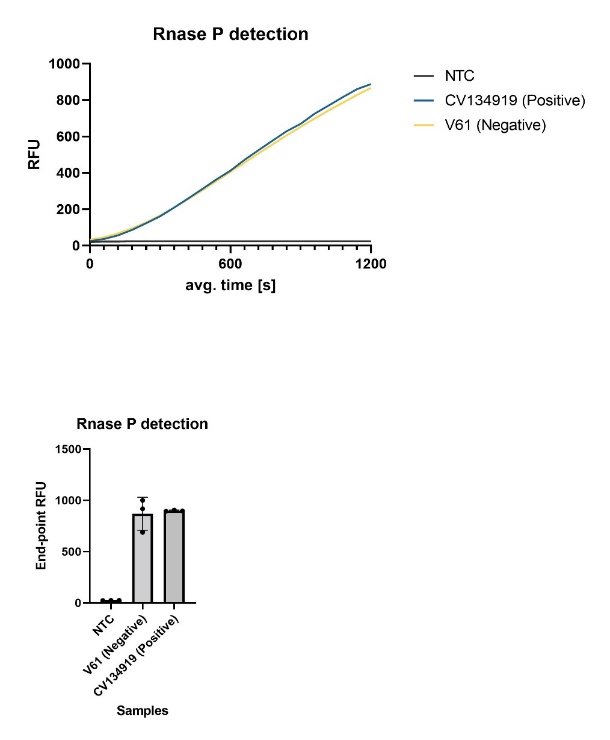

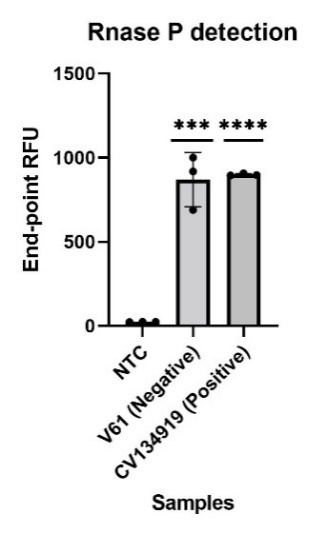


**Figure A4**. Clinical samples that were suspected to be positive or only positive for a specific set of genes were analysed via real-time RT-LAMP with a specific melting curve. (a). Melting curve analysis after the RT-LAMP reaction using the clinical samples that tested negative. (b). Individual gene melting curve analysis for sample CV134925 that tested negative in other tests, there is a very small amount of amplified genetic material for the N gene. (c) Enzyme kinetics for the Cas12a mixture with the Rnase P gRNA. (d) Validation of the internal control Rnase P to confirm the correct extraction of the genetic material. Statistical significance was determined by unpaired two-tailed t-test and all data were shown as mean ± S.D of 3 replicates. Asterisks indicate ** P<0.01; *** P<0.001; **** P<0.0001 and “ns” is non-significant.

In the end, the 3 samples that were considered negative were positive and thus the total amount of positive samples was 28 and the total amount of negative samples was 47 as represented in Table 1. Samples ranging from Ct 9 to a Ct of 37 were tested with excellent overall agreement and specificity for Ct below 35. Not all samples could be re-tested both for the Cas12a assay as well as for the real time RT-LAMP detection due to the low volume of extracted RNA. Despite this, the sensitivity of this test is almost at the threshold of the gold-standard where usually around cycles thresholds above 37, samples are considered negative for the virus.
